# Fabrication of Luminescent Microtiterplate Using Terbium Complex for Phenol Screening in Seawater Samples

**DOI:** 10.1007/s10895-024-03639-3

**Published:** 2024-03-08

**Authors:** Rasha M. Kamel

**Affiliations:** Faculty of Science, Chemistry Department, Suez University, Suez, 43518 Egypt

**Keywords:** Fluorescence, Terbium, Detection, Phenol, Pollution, Complex

## Abstract

Tb(III)-2-aminoterphthalate complex Tb_2_-(ATPh)_3_ was synthesized and characterized using FT-IR, thermal analysis and elemental analysis. Tb_2_(ATPh)_3_ microtiter plate was fabricated through embedding Tb(III) complex in polyvinyl chloride membrane and used for environmental determination of phenol in sea water samples. The calculated detection (*D*_*L*_), quantification (*Q*_*L*_) limits, and binding constant (*K*_*D*_) were 00.63 µmol L^− 1^, 2.10 µmol L^− 1^ and 1.32 × 10^4^ mol^− 1^ L, respectively. The fabricated microtiter plates exhibited high selectivity towards phenol over other hydrocarbon compounds. Furthermore, AGREE metric tool was used to assess the method’s green nature as well as its practicability and applicability. These merit outcomes provide that the new method for phenol detection was environmentally benign and safe to humans. The prepared Tb_2_(ATPh)_3_ MTP was validated through using gas chromatography for monitoring phenol in Suez Bay water accurately with high precision. The obtained results encouraged using Tb_2_(ATPh)_3_ MTP for efficient, fast, selective, and direct screening of phenol in real samples.

**Supplementary Information:**

The online version contains supplementary material available at 10.1007/s10895-024-03639-3.

## Introduction

The primary sources of phenols in seawater are the industrial and agricultural operations that release polluted effluent into the water. When phenolic compounds are exposed to water, they may change into new moieties that may be more hazardous than the original molecules [1]. These substances have the potential to harm both humans and animals and are poisonous and carcinogenic [2]. Phenolic compounds have been placed to the list of priority pollutants by the European Union and the United States Environmental Protection Agency (USEPA) [3–5].

First, phenolic compounds are extracted using solvent extraction, solid-phase extraction (SPE), or steam distillation extraction. Next, the compounds are determined using a variety of techniques, including GC-MS spectrometry, HPLC, capillary electrophoresis, and spectrophotometric techniques. These procedures take a lot of time and solvent; they are also complicated and need expensive equipment [6–14]. While fluorescence sensing [15–18] is distinguished by its ease of use, sensitivity, selectivity, dependability, low cost, and potential for creation of portable sensors.

A high-throughput method for environmental tests that sensitively quantifies trace analyte concentrations is luminescence detection in microtiter plates (MTP) format [19–25]. A strong statistical analysis of the fluorescence data is made possible by the sensing plate, which also allows for timesaving 8–12 channel parallelized sample preparation. Lanthanide complexes are being used increasingly frequently as fluorescence sensors due to their selectivity towards analytes. Sensing is accomplished by observing the intensity of lanthanide ion’s hypersensitive transitions [26–29].

Tb_2_(ATPh)_3_ microtiter plates (MTP) have been used to detect phenol in acetonitrile. It was obvious that phenol enhancing the intensity of hypersensitive peak of Tb_2_(ATPh)_3_ MTP. So, we employed Tb_2_(ATPh)_3_ MTP for an easily, accurate, and sensitive fluorometric screening of phenols in seawater.

## Experiments

### Materials

TbCl_3_·6H_2_O, phenol, 2-Aminoterephthalic acid (ATPh) and all solvents used were of ≥ 99.0%, purchased from Sigma Chemical Co.

### Apparatus

The UV/Vis, fluorescence measurements, elemental, FT-IR and thermal analysis were measured using Shimadzu-1601PC, JASCO FP8300 spectrofluorimeter, CHN-rapid analyzer (Heraus), BRUKER (ALPHA II) and Shimadzu H-60. Percent of Tb(III) in the complex was determined through EDTA titration with xylenol orange in acetate buffer.

### Synthesis of Tb(III)-2-Aminoterphthalate Complex

In a prior investigation, the Tb(III)-2-aminoterphthalate complex was already produced [30]. Dropwise addition of TbCl_3_.6H_2_O (1 mmol, 0.1687 g) to an ammoniated solution containing 2-aminoterphthalic acid (1 mmol, 0.0906 g) at 80–90 ^o^C with continuous stirring for one hour. The pale-yellow precipitate was filtered, repeatedly washed in hot water, and dried. CHN found (Calc.) C: 27.5 (28.8), H: 3.11 (3.10) N: 4.85 (4.21) and Tb % was 30.74 (31.82).

### Fabrication of Sensing MTP

The methods used to make MTP were the same as those described in our earlier research, which involved dissolving 0.5 g of polyvinyl chloride (PVC) polymer in 20 ml of DMF then, 20 mg Tb_2_(ATPh)_3_ complex dissolved in minimum volume of DMSO was added with strong stirring [25].

### Detection of Phenol Using Tb_2_(ATPh)_3_ Sensor

Fabricated MTP had 96 well, each on had filled with 50 µL of phenol (1 × 10^− 3^ mol L^− 1^) before 300 µL acetonitrile was added. By detecting change in the intensity of Tb(III) at λ_em_ = 545 nm and λ_exc_ = 350 nm using microtiter plate reader (FMP-125, JASCO, JAPAN) the sensing process is observed.

### Calculations

The binding constant (K) was determined using Benesi-Hildebrand equation [31].

$$\frac{1}{F-F^\circ }= \propto + \frac{\propto }{K \left[Phenol\right]}, \propto = \frac{1}{{F}_{L}-F^\circ } \left(1\right)$$

Where [phenol] denotes phenol concentration, F^o^ and F denote the intensity at λ_em_ = 545 nm in the absence and presence of phenol, respectively. F_L_ is the limiting intensity of fluorescence.

The calibration plot was used to determine the detection limit (LOD = 3σ/slope) and the quantitation limit (LOQ = 10 σ/slope).

While the following equation is used to compute the recovery (%) [25]:

$$Recovery \left(\%\right)=\frac{{S}_{Spiked} }{{R}_{Real}}x 100 \left(2\right)$$

Where S_Spiked_ and R_Real_ are concentration of phenol in spiked and real samples, respectively.

The quantum yield (QY) of Tb_2_(ATPh)_3_ complex was calculated using coumarine 6 (QY = 0.78 in ethanol) as a reference [24]. While the brightness equals to (ε. QY), where ε is the molar absorbance of the complex.

## Results & Discussion

### Tb_2_(ATPh)_3_ Solid Complex Characterization

The FT-IR spectrum of 2-aminoterphthalic acid is seen in **SI: Fig. ****S1**. During complex formation characteristic bands at 1690 cm^− 1^ for the protonated carboxylate groups is shifted to 1620 cm^− 1^ which indicated coordination of Tb^3+^ ion through one of the carboxylate group. The IR spectra of complexes show broad absorption bands in 3000–3600 cm^− 1^ range, belong to the OH group which confirm the presence of water molecules linked by hydrogen bonds in complex. The absence of NH_2_ symmetric and asymmetric stretches indicating the interaction between the amino group and Tb^3+^ ion [32].

Thermal stability over 400 ^o^C is a characteristic of the Tb(III)_2_-(ATPh)_3_ complex (**SI: Fig. S2**). The thermal analysis predicts that the M:L ratio for Tb(III)-2-aminoterphthalate complex is 2:3. Since the amino group of 4-aminoterephthatic acid did not coordinate any metal ions and only carboxylate groups participate in metal bonding, it encourages the creation of hydrogen bonds with water molecules. The Tb_2_(C_8_H_5_O_4_N)_3_.6H_2_O complex has a coordination number of eight for each terbium ion thanks to two oxygen atoms from water molecules and six oxygen atoms from the organic ligand, with four water molecules outside the coordination sphere [30]. Four hydrated water molecules are removed during the first breakdown stage, which terminates at 100 ^o^C and results in a mass loss of 7.78%, matching the theoretical value of 7.20.

### UV-vis and Fluorescence Spectroscopy of Tb_2_(ATPh)_3_ Sensor

**Fig. 1:**
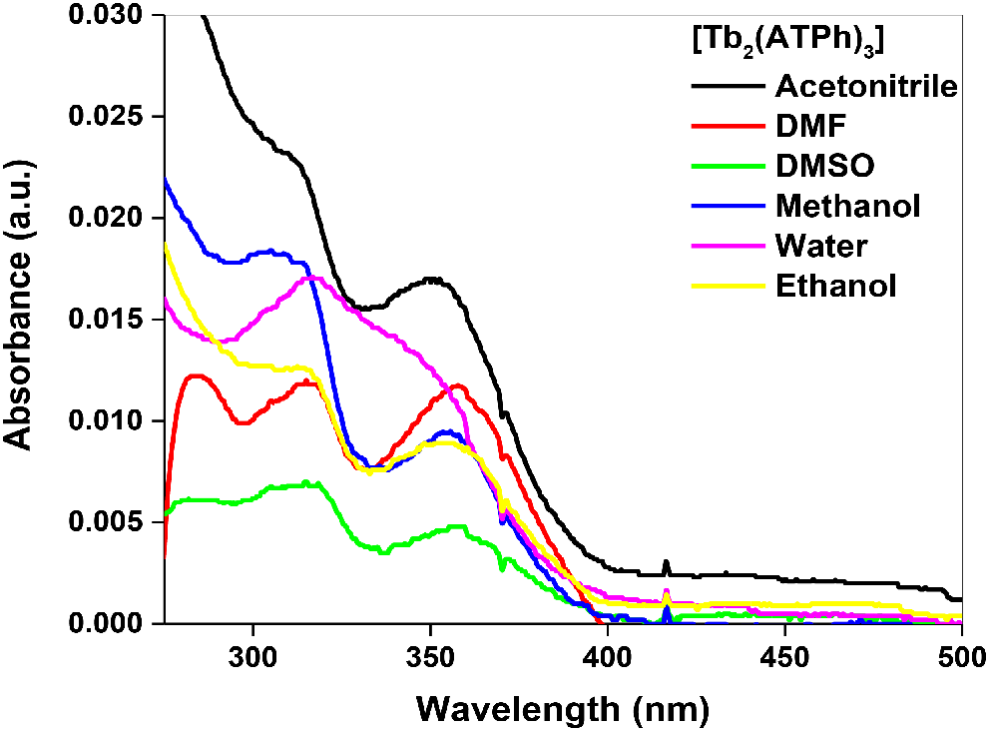
UV-absorption spectra for Tb_2_-(ATPh)_3_ complex

**Fig. 2:**
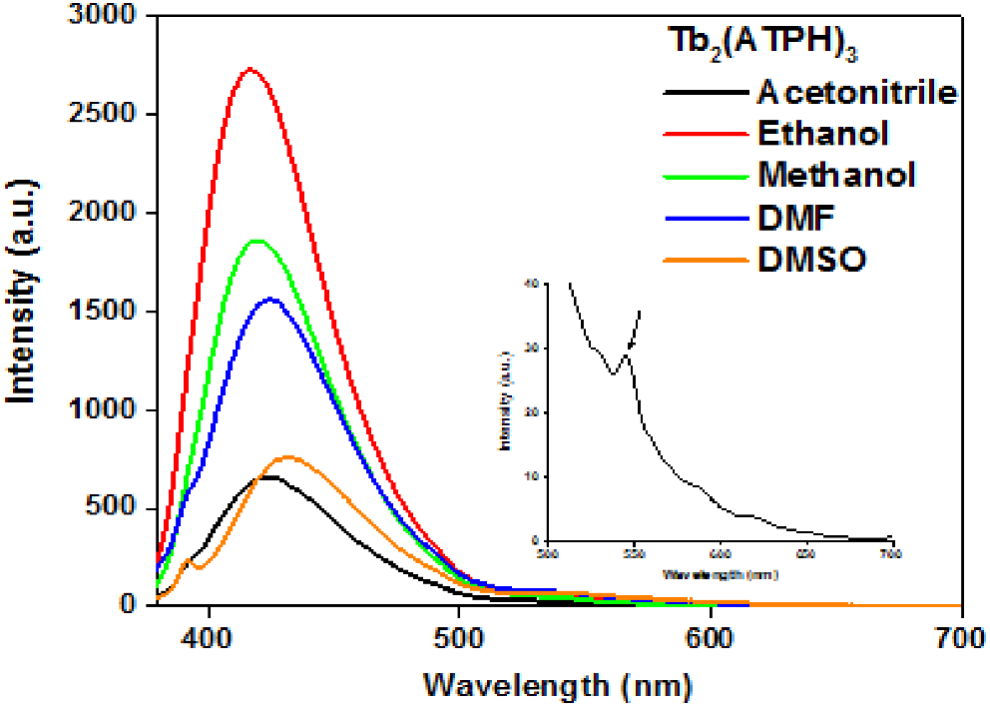
Emission spectra at λ_ex_ = 350 nm in different solvents at 25^o^C. Tb_2_(ATPh)_3_ complex in a concentration of 8 × 10^− 6^ mol L^− 1^

The UV-vis spectrum of Tb(III)_2_-(ATPh)_3_ (Fig. 1) exhibited a blue shift from 392 nm to 357 nm (~ 35 nm) for the π → π* transition of 2-aminoterephthalic acid due to the deprotonation of carboxylic acid and its binding to Tb^3+^ metal ions [32, 33] The quantum yield and brightness of Tb(III)_2_-(ATPh)_3_ complex was 0.045 and 27.0, respectively. After excitation at 350 nm, the emission spectra of Tb_2_-(ATPh)_3_ complex was measured and displayed a broad band between 390 and 500 nm which can be attributed to the excited states of 2-aminoterephthalic acid moiety. Moreover, the fluorescence spectrum showed also weak peak at λ_em_ = 545 nm characteristic to Tb(III) (^5^D_4_→^7^F_5_ transition) in acetonitrile only while the characteristic emission peaks not observed in other solvents (Fig. 2), so the further studies were examined in acetonitrile.

nm in different solvents at 25^o^C. Tb_2_(ATPh)_3_ complex in a concentration of 8 × 10^− 6^ mol L^− 1^.

### Using of Tb_2_(ATPh)_3_ Sensor for Phenol Detection

**Fig. 3:**
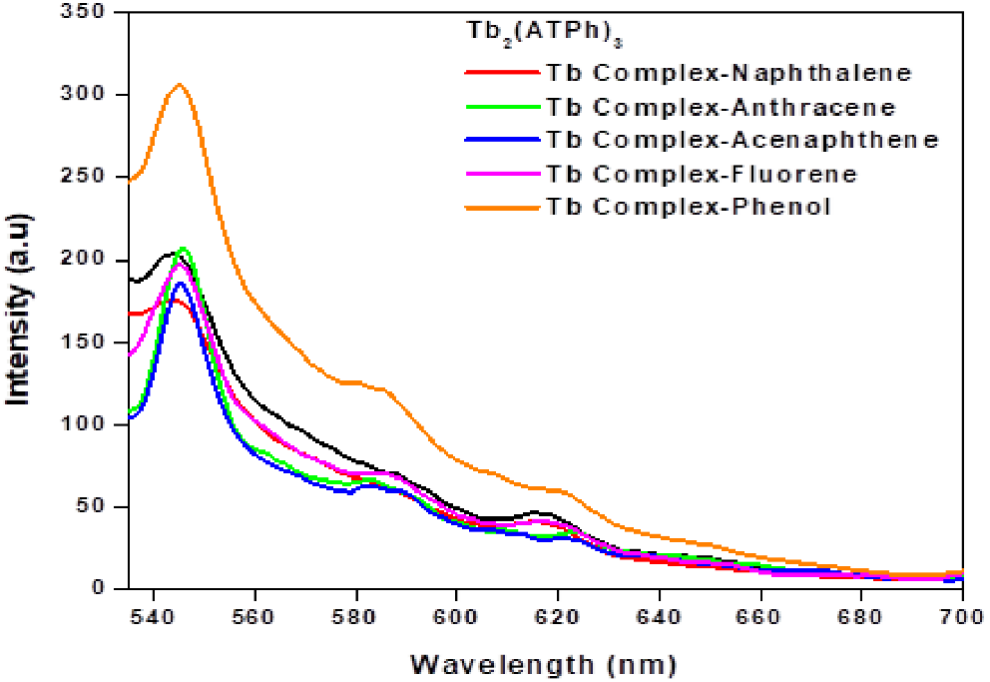
Emission spectra for the interaction of Tb_2_(ATPh)_3_ complex with different PAHs (20 µmol L^− 1^) in acetonitrile, λ_ex_ = 350 nm and λ_em_ = 545 nm at 25^o^C

**Fig. 4:**
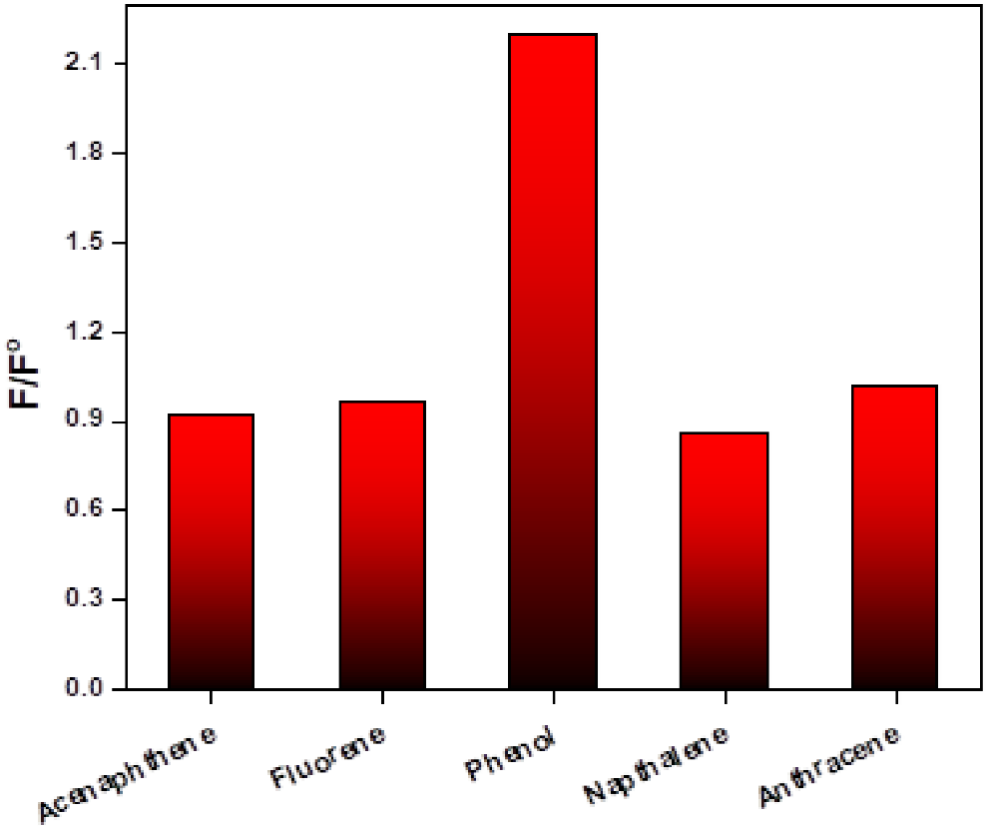
F/F^o^ ratio of the luminescence intensity of Tb_2_(ATPh)_3_ in presence of 10 µmol L^− 1^ of different testing species in acetonitrile, λ_ex_ = 350 nm

The addition of 10 µmol L^− 1^ of different polycyclic aromatic hydrocarbons (PAHs) (anthracene, fluorene, naphthalene, acenaphthene and phenol) to 10 µmol L^− 1^ Tb_2_(ATPh)_3_ solution have been investigated in acetonitrile. It was noticed that the addition of 10 µmol L^− 1^ phenol on Tb_2_(ATPh)_3_ complex solution enhancing the fluorescence intensity at λ_em_ = 545 nm of the complex as shown in Fig. 3, otherwise the other hydrocarbons cause no significant change. The F/F^o^ values for different PAHs is depicted in Fig. 4, where F^o^ and F denoted to the emission intensity of the Tb_2_(ATPh)_3_ complex in absence and presence of 10 µmol L^− 1^ of different PAHs. The F/F^o^ value is around 1 for all PAHs but has a higher value than 1 for phenol. Therefore, phenol is selected for further detailed investigation, i.e. the probe Tb_2_(ATPh)_3_ complex can be used as selective probe for phenol.

**Fig. 5:**
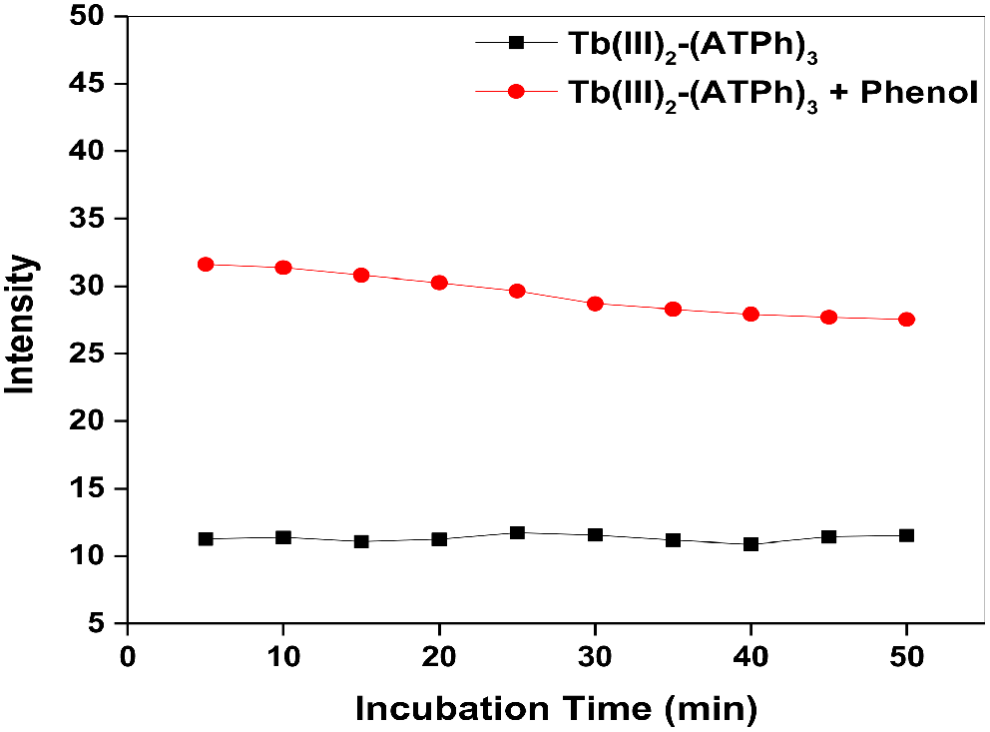
Plot of incubation time on intensity of Tb complex MTP peak at λ_em_ = 545 nm

The solid fluorescence spectrum of MIP was examined; the λ_exc_ = 350 nm, while λ_em_ = 545 nm corresponding to ^5^D_4_→^7^F_5_ transition (**SI: Fig. S3**). The incubation time of Tb_2_(ATPh)_3_ MTP means the time necessary for obtaining the steady signal emission. It can be seen in Fig. 5 that the incubation time of Tb_2_(ATPh)_3_ MTP for phenol detection was 10 min.

#### Calibration Plot

**Fig. 6:**
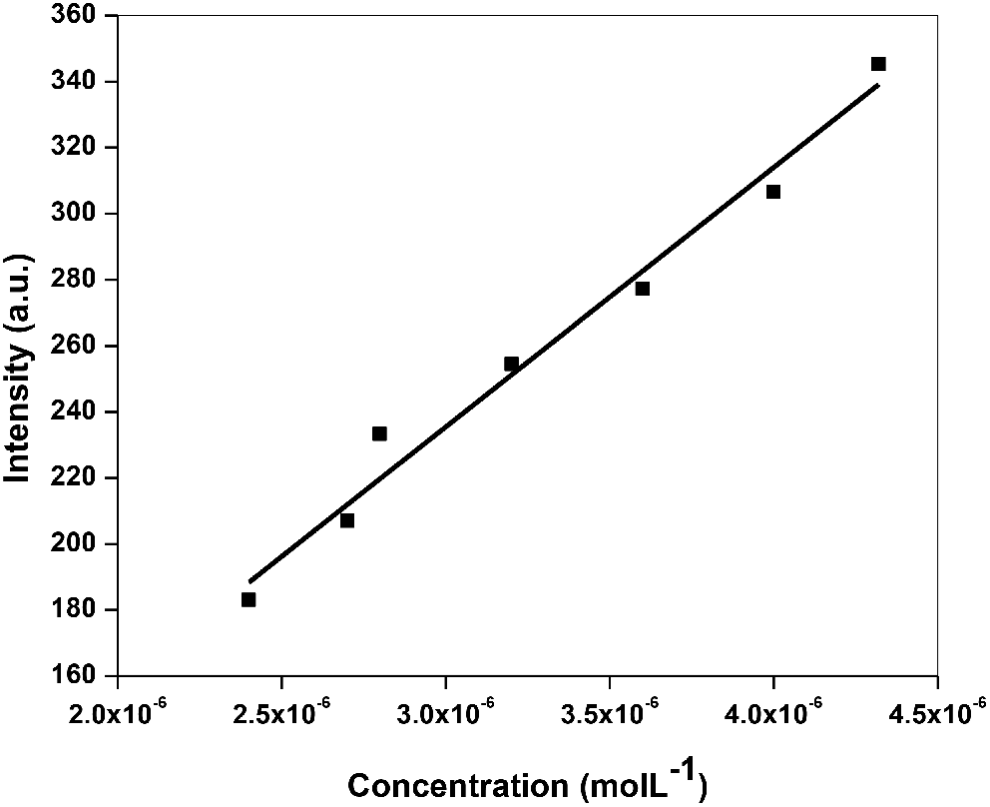
Calibration plot for the interaction of Tb_2_(ATPh)_3_ MTP with phenol in a concentration range 2–20 µmol L^− 1^ at λ_ex_ = 350 nm and λ_em_ = 545 nm

The increasing phenol concentration in the range of 0.2 to 20 µmol L^− 1^ in acetonitrile has a significant effect on the emission spectrum of Tb_2_(ATPh)_3_. By increasing the concentration of phenol, the hypersensitive emission peak intensity for Tb_2_(ATPh)_3_ complex (λ_em_ = 545 nm) was greatly increased. A calibration plot of different concentrations of phenol with the Tb_2_(ATPh)_3_ complex was obtained (Fig. 6). The limit of detection (*D*_*L*_) and limit of quantification (*Q*_*L*_) equal to 0.63 µmol L^− 1^ and 2.10 µmol L^− 1^, respectively. The calibration parameters are mentioned in Table 1. The developed sensor has demonstrated a very good binding affinity to phenol, as indicated by the high value of the binding constant (*K*_*D*_). (**SI: Fig. S4**). The *D*_*L*_ of phenol obtained using Tb_2_(ATPh)_3_ MTP was compared with those of previous studies (Table 2) [34–40].

**Table 1:** Calibration parameter for determination of phenol using Tb_2_(ATPh)_3_ MTP

**Parameters**	**Phenol**
LOD (µmol L^− 1^)	0.63
LOQ (µmol L^− 1^)	2.10
Linearity range (µM)	0.2–5
Standard deviation, *n* = 7	16.48
Regression coefficient	0.9769
Slope	7.85 × 10^7^
Binding Constant (*K*_*D*_) (mol^− 1^ L)	1.32 × 10^4^

**Table 2:** Comparison between Tb_2_(ATPh)_3_ MTP and the reported methods for phenol detection

**Sensor**	**Detection Limit**	**Ref.**
Interaction of phenol with 2,6- Dichloroquinone-4-chloroimide	1.0 µmol L^− 1^	35
Interaction of the enzymatic oxidation product of the phenolic compound with 3-methyl-2-benzothiazoline hydrazine (enzymes: peroxidase, laccase, and tyrosinase)	5.0 µmol L^− 1^	36
Single-stranded DNA (ssDNA)-regulated gold nanoparticles (AuNPs) as indicators	1.6 µmol L^− 1^	37
The tyrosinase enzyme has been immobilized on a filter paper by simple over-spotting with 3-methyl-2-benzothiazolinone hydrazone (MBTH)	5.0 µmol L^− 1^	38
Molecularly imprinted polymer membranes obtained by co polymerization of the complex Cu(II)–catechol–urocanic acid ethyl ester with (tri)ethyleneglycoldimethacrylate, and oligourethaneacrylate	63 µmol L^− 1^	39
naked Fe_3_O_4_, PEG-coated Fe_3_O_4_, and CTAB-coated Fe_3_O_4_ magnetic nanoparticles (MNPs).	3.0 µmol L^− 1^	40
Amperometric / colorimetric biosensor	1.0 µmol L^− 1^	41
Tb_2_(ATPh)_3_ MTP	0.63 µmol L^− 1^	Our work

**Fig. 7:**
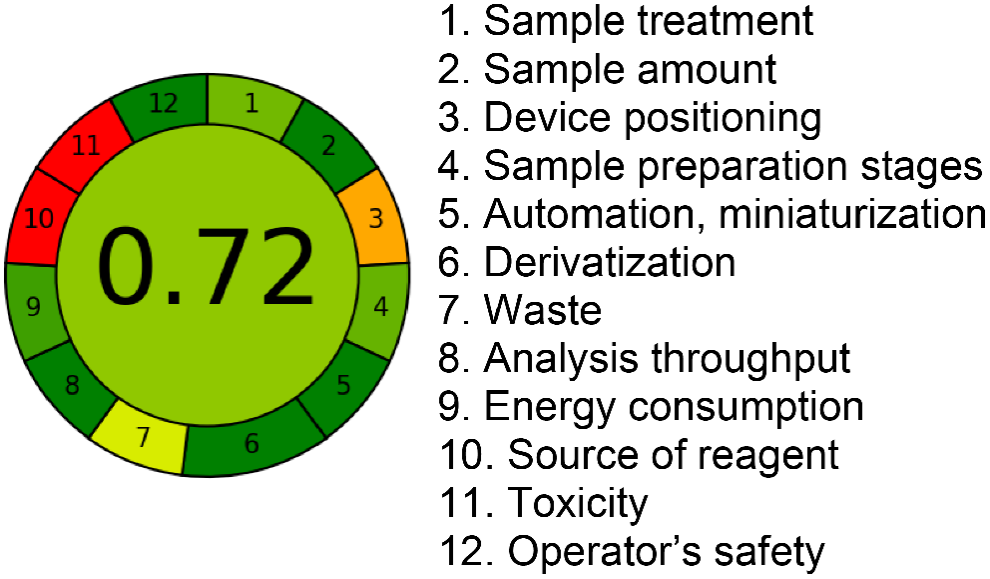
AGREE Assessment for the analysis of phenol using Tb_2_(ATPh)_3_ MTP

Evaluation of Greenness Attributes with the new microtiter plate reader technique. The AGREE approach efficiently evaluates the efficacy and reliability of greenness attributes [41, 42]. The developed microtiter plate gave an AGREE score of 0.72, indicating the relative greenness attributes of the Tb_2_(ATPh)_3_ microtiter plate for phenol detection, (Fig. 7). Furthermore, the somewhat increased AGREE value (0.72) can be attributed to specific factors that were concluded in the use of the microplate format, which is designed to analyze 96 samples in each run and consumes a very low amount of sample (150 µL) and solvent (300 µL), further enabling time savings (1 min for each run). While red subsectors 10, and 11 are attributed to the source of solvents and their toxicity, respectively. The use of these solvents is necessary for the extraction and preconcentration of phenol.

#### Interference Study

The selectivity of the proposed method using Tb_2_(ATPh)_3_ MTP in the detection of phenol has been tested against several interferents that may interfere with the detection method. Table 3 shows the tolerance concentration of interferents during the determination of 10 µmol L^− 1^ of phenol. The results demonstrate the selectivity of Tb_2_(ATPh)_3_ MTP for the detection of phenol.

**Table 3:** Influence of interfering species and their tolerated concentrations

**Tolerance limit for foreign species (µmol/L)**									
**K** ^**+**^	**Na** ^**+**^	**Cu** ^**2+**^	**Co** ^**2+**^	**Ni** ^**2+**^	**Al** ^**3+**^	**Acenaphthene**	**Anthracene**	**Naphthalene**	**Fluorene**
200	200	100	100	100	100	50	40	40	65

#### Recovery

The recovery investigation was conducted by introducing a specific quantity of phenol to different water samples (tap, seawater, and agriculture wastewater). The RSD values were in the range of 0.57 to 3.52%. Table 4 demonstrates the high precision and reproducibility of the developed method based on Tb_2_(ATPh)_3_ MTP.

**Table 4:** Recovering results for phenol in spiked tap, seawater, and agriculture wastewater samples

**Type of sample**	**Added (µmolL** ^**− 1**^ **)**	**Found (µmolL** ^**− 1**^ **)**	**Recovery (%)**	**RSD (n = 3)**
**Tap water**	1	1.03	97.09	14.78
	3	2.90	103.45	3.45
	5	5.07	98.62	2.28
	10	9.97	100.30	3.52
	15	15.17	98.88	1.01
**Sea water**	1	1.17	85.47	4.95
	3	3.10	96.77	3.23
	5 10	5.17 10.07	96.71 99.30	2.96 0.57
	15	15.13	99.14	0.76
**Agriculture wastewater**	1	0.97	103.05	11.95
	3	3.03	99.00	1.90
	5	5.07	98.62	2.28
	10	9.90	101.01	2.67
	15	15.13	99.14	0.76

#### Analysis of Phenol in Real Samples Using Tb_2_(ATPh)_3_ MTP

The samples of seawater were taken from various parts of the Gulf of Suez. Employing the APHA extraction procedures to extract phenol from seawater samples, then a Hewlett-Packard 5890 Series II gas chromatography (GC) model was used to evaluate the amounts of phenol in samples of seawater [43]. The phenol concentration was quantitatively measured using Tb_2_(ATPh)_3_ MTP. It was found that the outcomes were quite consistent with the gas chromatography data (Table 5).

**Table 5:** Detection of phenol in wastewater samples using Tb_2_(ATPh)_3_ (MTP)

**Sample**	**Concentration of proposed method (µmolL** ^**− 1**^ **)**	
	**Tb**_**2**_ **(ATPh)**_**3**_ **MTP**	**GC Method**
Sample 1	0.96	0.98
Sample 2	8.01	8.13
Sample 3	11.38	11.45

Sample 1 collected from Abu sultan Electrical Steam Power Station

Sample 2, 3 collected from Suez Oil Processing Company

## Conclusion

Tb_2_(ATPh)_3_ MTP was developed and applied for fluorometric detection of phenol in seawater samples. Our new method involves recording the enhancement of the hypersensitive emission peak of Tb(III) at 545 nm. The MTP preserves time and effort as it enables rapid and simultaneous detection of up to 96 (real) samples, so we can screen many samples in short time. Additionally, Tb_2_(ATPh)_3_ MTP provided high sensitivity and selectivity towards phenol over other interferent compounds.

## Electronic Supplementary Material

Below is the link to the electronic supplementary material.

Supplementary Material 1

## Data Availability

No datasets were generated or analysed during the current study.
