# Supplementary material for: Fabrication of Luminescent Microtiterplate Using Terbium Complex for Phenol Screening in Seawater Samples
Source: J Fluoresc. 2024 Mar 8;35(3):1833–9. doi: 10.1007/s10895-024-03639-3 (PMC11968531; doi:10.1007/s10895-024-03639-3)
Supplement: Supplementary file 1 — Supplementary Material 1 [file 10895_2024_3639_MOESM1_ESM.docx]

**Supplementary Information**

**
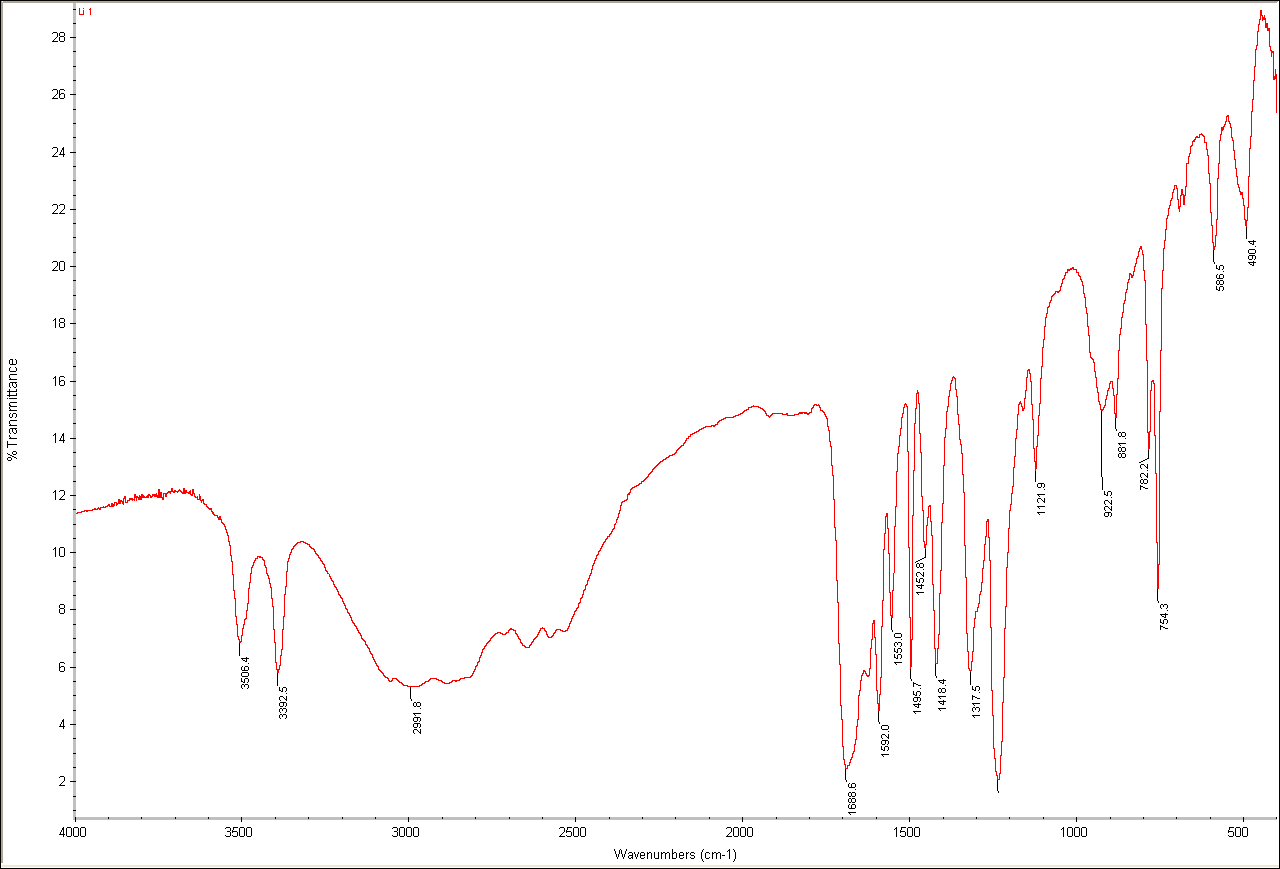
**

**A**

**
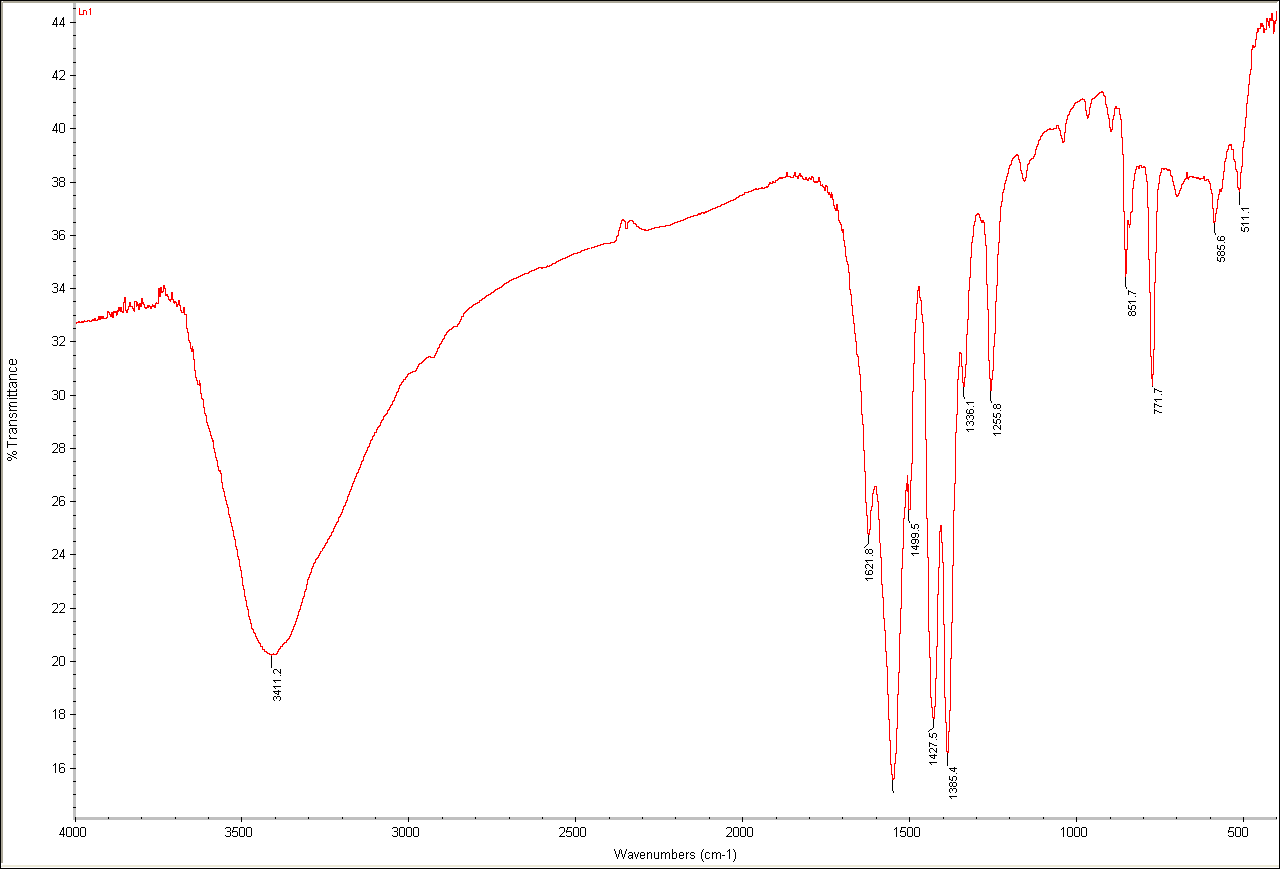
**

**B**

**SI: Fig. S1:** FT-IR spectrum of 2-amniophthalic acid (**A**) and Tb(III) complex (**B**)


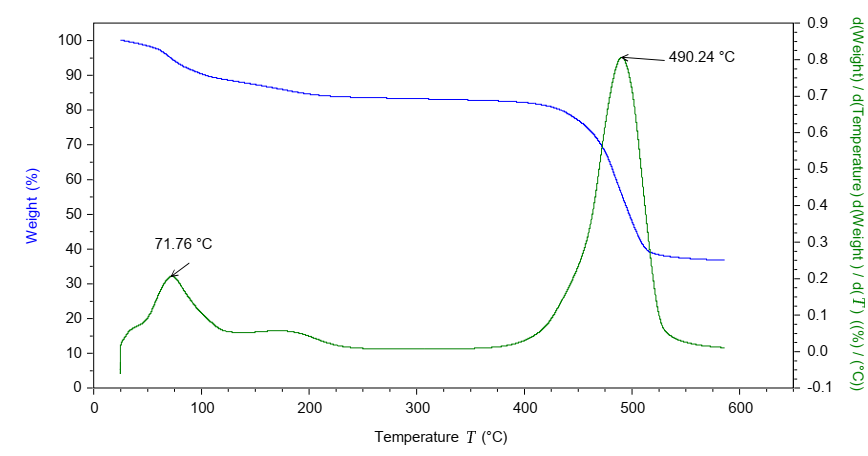


**SI: Fig. S2:** Thermogram of Tb(III) complex

**SI: Fig. S3**: solid emission spectra at λ_ex_ = 350 nm of Tb_2_(ATPh)_3_ MTP


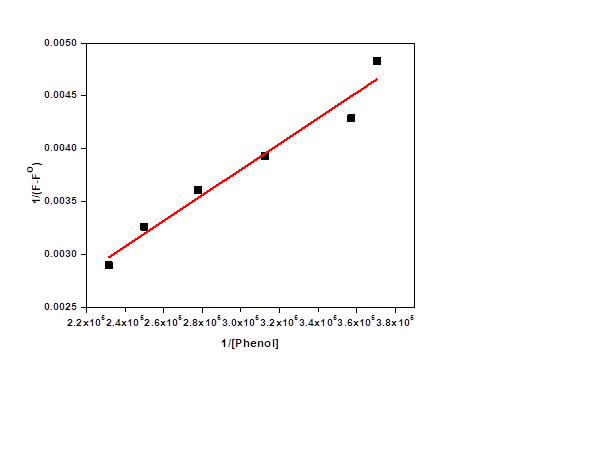


**SI: Fig. S4:** Benesi-Hildebrand relation for the interaction of different concentrations of phenol with Tb_2_(ATPh)_3_ MTP, in acetonitrile (λ_ex_= 350 nm).
